# Patients Receiving Palliative Care and Their Experiences of Encounters With Healthcare Professionals

**DOI:** 10.1177/00302228221077486

**Published:** 2022-03-02

**Authors:** Haavisto Elina, Eriksson Sofia, Cleland Silva Tricia, Koivisto Jaana-Maija, Kausamo Katariina, Soikkeli-Jalonen Anu

**Affiliations:** 1 Health Sciences Unit, Faculty of Social Sciences, 7840 Tampere University, Finland; 2 Department of Nursing Science, 8058 University of Turku, Finland; 3 Satakunta Central Hospital, Pori, Finland; 4 Finno-Ugrian and Scandinavian Studies, 101230 Faculty of Arts, University of Helsinki, Finland; 5 3856 Hanken School of Economics, Helsinki, Finland

**Keywords:** palliative care, end-of-life, encounter, hospital care, patient, cancer

## Abstract

The study aimed to explore experiences of encounters with health care professionals among patients receiving palliative cancer care in specialist palliative care inpatient units. A qualitative explorative study design was conducted in a specialist palliative care inpatient setting. Data collection was implemented using semi-structured individual interviews (20 palliative care cancer patients) and analysed with inductive content analysis. Palliative care patients experienced both meaningful and disrespectful encounters with healthcare professionals. The meaningful encounters encompassed authentic and supportive experiences, while the disrespectful encounters included indifferent and inadequate experiences. Caring for a patient receiving palliative care requires care beyond tending to a patient’s physical needs. Patients should be encountered holistically and as equal human beings without highlighting their roles as patients. The healthcare professionals and the organisations should also acknowledge the importance of time and effort spent for encounters and conversations with the patients instead of concentrating resources mainly on physical care.

## Introduction

The world’s population is ageing, which generates increasing demand for palliative care services. Every year about 50 million people need palliative care worldwide (Connor, 2020). Globally, every one to six deaths is caused by cancer, and cancer patients form a large part of palliative care patients (WPCA & WHO, 2014). In addition, several studies have highlighted the need for quality palliative and end-of-life care (Hales, 2008).

Human interaction includes encounters that can be defined as honest and open communication, in which people allow themselves to be seen in all their vulnerability (Rock, 2019). Encounters in healthcare can be characterised as caring encounters with healthcare professionals and patients requiring presence, recognition, availability and mutuality (Holopainen et al., 2019). Moreover, caring encounters include openness, patience, empathy, communication and sensitivity (Wälivaara et al., 2013). Patients need to be treated respectfully, through active listening of the healthcare professionals, for the patient to perceive the encounter as positive (Nygårdh et al., 2012). Patients expect personnel to have sensitivity, openness and attentiveness, in addition to cognitive and clinical knowledge (Timmermann et al., 2017). Healthcare professionals are supposed to offer emotional care, in addition to physical care (Chua et al., 2020). From the healthcare professionals’ point of view, a good encounter means being personal and professional at the same time (Wälivaara et al., 2013).

Good caring encounters can be seen as individual, person-to-person connections in which the interaction happens as equals (Wälivaara et al., 2013). Patients with life-threatening conditions need to be treated as unique and whole people, not just as a case of ‘diseases’ (Söderman et al., 2020). Healthcare professionals’ ethical awareness is needed to ensure successful encounters, quality of care (Akin Korhan et al., 2018) and possibilities for the patient to make autonomous decisions regarding their regular care, all of which requires frequent conversations with the healthcare professionals (Houska & Loučka, 2019; Nygren Zotterman et al., 2016). The care of dying people also requires a capability to appreciate the uniqueness of the caring situations to handle the everyday encounters and care with dignity (Haavisto et al., 2020). However, studies have shown that attitudes towards palliative care patients are not always holistic (Akin Korhan et al., 2018; Kmetec et al., 2020; Price et al., 2019) and physical needs are prioritised over patients’ emotional and spiritual needs (Kisvetrová et al., 2017; Zweers et al., 2019).

While good caring encounters can enhance patients’ positive experiences of care, uncaring encounters may lead patients to feel that the care they have received is deficient and unempathetic (Cheruiyot & Brysiewicz, 2019). Uncaring encounters can cause negative emotions through the objectification and dehumanisation of the patient (Hemberg & Bergdahl, 2020; Holopainen et al., 2019). If patients are treated with carelessness, distance and nonchalance, and if there are not enough opportunities for face-to-face conversations, patients can feel meaningless and insignificant, devoid of their humanness (Nygren Zotterman et al., 2015). Feelings of being ignored or treated with indifference affect the patient’s experience of dignity, which might impact their health and well-being (Skär & Söderberg, 2018). Patients can feel excluded if they believe their fundamental needs are not being met (Jørgensen et al., 2019).

Studies about encounters vary by content, and patient encounters are usually described from the healthcare professionals’ views, mostly physicians and nurses (Holopainen et al., 2019), and studies from the patients’ perspective are rare. Thus, this study aims to explore experiences of encounters with health care professionals among patients receiving palliative cancer care in specialist palliative care inpatient units. By identifying the kinds of encounters, a deeper understanding can be obtained of the significance of the palliative care environment for patients and potentially improve patients’ quality of care (Skär & Söderberg, 2018).

## Method

### Study Design

A qualitative explorative study (Rendle et al., 2019) was performed on semi-structured individual interviews and inductive content analysis (Elo et al., 2014). A consolidated criteria for reporting qualitative studies (COREQ), 32-item checklist, was utilised during the analysis and reporting (Tong et al., 2007).

### Participants

The study focuses on purposely selected interviewees who have had experiences in palliative hospital care (Elo et al., 2014). The participants were 20 patients receiving palliative care in specialist inpatient units in four hospitals of two large university hospital districts in Finland. Specialist palliative care is provided in care units where the professionals have particular expertise in palliative care (Forbat et al., 2020). In Finland, specialist palliative care is concentrated in particular units where the main task is organising palliative and end-of-life care. Therefore, it has been recommended that the care personnel have special palliative care education (Saarto, 2019). The inclusion criteria were as follows: patients ≥18 years old, Finnish-speaking, had received palliative hospital care for at least 1 week, were incurably ill and had received only symptomatic treatment and fundamental care. In addition, the patients had to be willing to participate and able to describe their individual experience of care. The suitability of each patient was assessed by a contact person, a registered nurse within the unit. All patients considered suitable and willing to participate were included.

### Collection of the Data

The data were collected from May to November 2019. Interviews were conducted either by a female research assistant working as a palliative care nurse or a female PhD student. Interviewers had no care relationship or other connection to the patients. Information about the interviewer’s position at the research project and research goals were given to the participants before the interviews. The interviews focused on three themes related to palliative care: symptom management, informative support and emotional support. The themes were constructed regarding the everyday activities and situations, which recur on a daily basis in palliative inpatient units and involve encounters based on previous literature (Borneman et al., 2015; Jangland et al., 2008; Johnston et al., 2015; Skär & Söderberg, 2018; Virdun et al., 2017; Wang et al., 2018). All interviews were conducted following similar predefined themes. Patients were asked to describe their experiences and give examples of the following: (1) symptom management, (2) informative support and (3) emotional support. The interviewer helped them identify the situations where encounters occur. That was important because of the condition of the palliative care patients whose strain in the interview situation was sought to be minimised. The interviews lasted an average of 23 minutes (9½–50 minutes) regarding the patient´s condition, and they were conducted privately in the patient rooms.

For most patients, the brief situation was the only opportunity to implement the interview, and the interviews could not be continued further or repeated. The interviews were audio recorded, and the data were collected until it saturated.

### Data Analysis

Data were analysed using inductive content analysis to uncover the meaning of lived experiences through individuals who have personal insight into the phenomena. Qualitative research, using inductive content analysis, enriches understanding of the studied phenomena and brings light to the crucial and prominent perspectives within healthcare (Elo et al., 2014). The interviews were analysed first by two researchers (xx and xx) and then discussed with a research team.

The data was transcribed verbatim and, after that, a unit of analysis was defined as a sentence that described the patient´s experience of the encounter. The sentences were then simplified and coded to enhance the analysis. The data were systematically analysed by returning to study the drafts and comparison, searching for connections and seeking possible patterns across the individual interviews. The findings of patterns were drafted to form overlapping categories. These categories were then reviewed and identified and drafted as sub-categories. Finally, these sub-categories were combined to create categories, including similar content, and after that, the main categories were established. An example of the analysis process is presented in Table 1.

**Table 1.** An Example of the Analysis Process.

Original Expression	Simplification	Subcategory	Category	Main Category
Encounter is the most important; patients are not objects ID1	Encounter is the most important Patients are not objects	Being seen as a human being	Authentic encounters	Meaningful encounters
There is good support here, and I have been listened to ID11	Good support here Has been listened to			
They ask how I am doing ID 8	They ask how I am doing			
Kind and unhurried, does not in any way indicate that now he is in a bit of a hurry ID5	Kind Unhurried Not in a hurry			
I have been asked just enough about my wishes for treatment ID8	Have been asked enough about my care wishes.	Experiences of autonomy		
My doctor helped me get home where I want to die ID8. I can be here for a long time, according to my well-being ID14	My doctor helped me get home where I want to die. I can be here for a long time, according to my well-being			
It is nice to have your own room and peace ID13	Nice to have your own room Nice to have peace			
The hospital is a boring place. The sauna was a chance to get away from the hospital world ID2	The sauna helps out of the hospital world			

### Ethics Considerations

Ethical approval was obtained from the Ethics committee of the University of Turku (15/2019). The participants’ autonomy, privacy and anonymity were respected at all stages of the study (National Library of Medicine, 2013). The interviews have been conducted in a calm place to ensure the confidentiality. The place was chosen by the interviewee. The patient was asked not to disclose her/his name or the names of family members. The participants’ right to self-determination was respected by informing them about the voluntary nature of participation and their right to interrupt the interview at any time. Written informed consent was obtained from all participants prior to the study. The mental and physical capacity and the condition of the patients were observed during the interviews. For instance, the interviewer observed the patient’s emotional state and fatigue, and if the patient seemed anxious or tired, the interviewer asked if the interview should be interrupted or stopped. The interviewees’ privacy and data protection rights were respected in the handling and storage of material (General Data Protection Regulation, 2016).

## Results

Of the patients interviewed, 13 were women. The average age of the patients was 75.3 years (58–94). Patients had 10 different types of cancer diagnoses. They had been suffering from their illness for an average of 2.8 years (2 months–17 years) and been in inpatient treatment for an average of 2.8 weeks (1–8 weeks).

### Experiences of Encounters Among Patients Receiving Palliative Cancer Care

**Figure 1.**
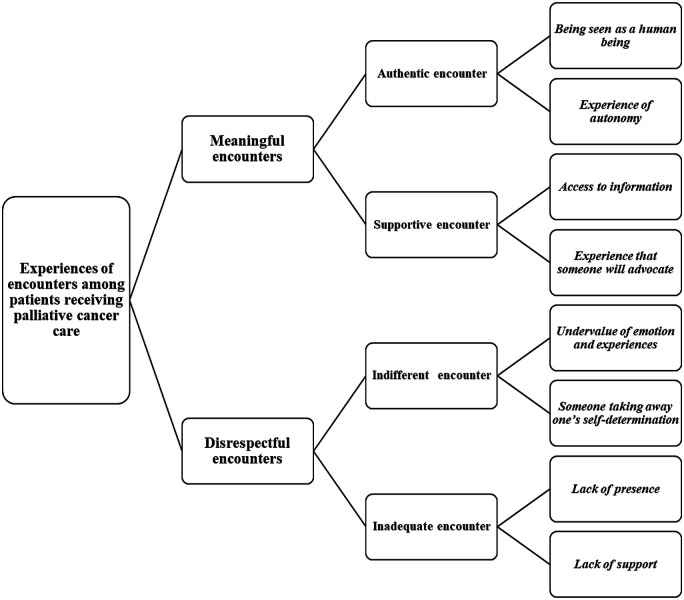
Meaningful and Disrespectful Encounters Experienced by Patients Receiving Palliative Cancer Care.

Experiences of encounters among patients receiving palliative cancer care were described through two main categories: meaningful encounters and disrespectful encounters with healthcare professionals (Figure 1).

### Meaningful Encounters with Healthcare Professionals

A meaningful encounter consisted of two categories: an authentic encounter and a supportive encounter.

#### Authentic Encounter

Several patients described a meaningful encounter to be one that is authentic. The experiences of an authentic encounter occured in two sub-categories: *being seen as a human being* and *the experience of autonomy.*

Patients described experiencing an authentic encounter as *being seen as a human being* rather than as a physical body to be treated, as one patient said: an ‘encounter is most important; patients are not objects’ ID1. An encounter was manifested in patients as the ability of health professionals to create dignity, supportive and empathic interactions that mediated life experience and interpersonal skills of the healthcare professionals. One patient told that ‘there is good support here, and I have been listened to’ ID11. Another patient described the staff as ‘warm and friendly…treated with dignity’ ID1. He also talked about the ability of staffs to show empathy and create positive encounters as, in his words, ‘it is more about life experience and personality than a degree’ ID1. A third participant told that ‘here dare to say things, not everywhere dare’ ID16. It was important for patients that the staff were interested in patients, and they had time to encounter them which two patients described as the following: ‘kind and unhurried, does not in any way indicate that now he is in a bit of a hurry’ ID15, and ‘they ask how I am doing’ ID 8. The patients felt that the staff allowed them to leave the patient role, among other things, by allowing smoking for those who wanted it. One patient said that ‘the cigarette breaks are the favorite part of my day, since that is where life comes together’ ID1.

Discussion on the future, as it had been before in their life, depicted an authentic encounter too. The patients described that their future should be considered as valuable as anyone else’s, even though it was redefined in the context of palliative care when having an incurable illness. One patient described the issues as ‘despite the fact that I´m going towards death, my future is just as valuable as any other human beings’ ID11. The conversation of death was also meaningful as three patients expressed as follow: ‘future information on what to think about the future… even though we are about to die’ ID11; ‘I have discussed with the doctor about what lies ahead’ ID2; and ‘I need to express fear, worry, and sadness related to my future’ ID18. What was common and crucial for these patients was finding their ways to relate to their coming passing. However, the interviews revealed that the future might be forgotten as one patient described ‘the patient’s future is easily forgotten here, even though we still have one’ ID11.

*The experience of autonomy* meant the possibility to influence personal care and respect for privacy. Many patients had a feeling that they could influence their care if they wished as one patient described, ‘I have been asked just enough about my wishes for treatment’ ID8. Another said that ‘I’ve been asked if I agree with this or that treatment … it’s wonderful to be able to have my say’ ID16. Although, if they did not have an opportunity to control their care, the feeling of having a possibility to influence was significant. According to the interviews, the individual care plan enabled patients’ wishes to choose their care environment such as moving back home, where some patients wanted to die rather than at the hospital. For instance, one patient described this experience as ‘my doctor helped me get home where I want to die’ ID8. It was important for some patients to know that they could stay in the hospital in the same ward for as long as they wanted which one patient expressed as this, ‘I can be here for a long time, according to my well-being’ ID14. Another told that ‘it feels good not to have to move from place to place’ ID13. Privacy, one’s peace, and the opportunity to be alone if desired were also associated with an experience of autonomy as one patient described, ‘it is nice to have your own room and peace’ ID13. (e.g. self-determined alone time in the sauna was a get-away from the hospital culture of being ill). The patients told that ‘the hospital is a boring place. The sauna was a chance to get away from the hospital world’ ID2.

### Supportive Encounter

A supportive encounter included two sub-categories: *access to information* and *experience that someone will advocate.*

A patient’s experience, *access to information*, meant, above all, that the healthcare professionals were aware of the patient’s need for information, that patients received information, and that the threshold to ask for information was low. The information received from the doctor about the overall situation regarding the disease helped patients to cope. One patient said that ‘the doctor has told me everything that happens, what happens in my body… otherwise, nothing would have come of it’ ID2. Another patient told that ‘I have been explained why I am not receiving treatment’ ID5. Patients trusted their physicians to know the level of information that was right for them. In addition, some patients had asked their physician to omit details that might be too overwhelming for the patient when discussing their illness, and some patients did not want information at all. In these cases, patients also said they protect themselves as one patient described ‘I have protected myself. I will enjoy this time that I can live, and I trust the doctors’ ID1. Patients described the possibility of changing their minds freely if they, at any time, wanted to be more thoroughly informed. ‘You can contact your doctor immediately if you have any questions’ ID5 as said by a patient.

*Experience that someone will advocate* meant efforts by patients’ physicians to organise suitable palliative care facilities for them as one patient described ‘my doctor arranged palliative care for me so that I could get into this ward’ ID2. Implementing the care plan to improve well-being showed the patients that healthcare professionals were on their side. One patient said that ‘the doctor is setting up a plan to rehabilitate my legs so that I could strengthen them and move back home’ ID7. The experience that healthcare professionals were on the patient’s side was also related to the fact that patients felt they could ask for as much help as they needed without apologising. Furthermore, patients had the experience that nurses had encouraged them to ask for help. One patient described the situation as ‘the nurse said we are here in the ward to help patients’ ID 18. Another said that ‘it has been said that I get as much morphine as I want, it makes you feel safe’ ID 4 and another told that ‘if there is pain, you can just say and get medicine’ ID8.

### Disrespectful Encounters with Healthcare Professionals

**Disrespectful** encounters with healthcare professionals comprised of two categories: indifferent *encounters and inadequate encounters* (Figure 1).

#### Indifferent Encounters

*The Indifferent encounters* are divided into two sub-categories: *undervalue emotion and experiences* and *someone taking away one’s self-determination*.

The patients described feelings that *their emotions and experiences were undervalued*. The patient’s feelings were not taken into account when talking about the prognosis of the disease as one patient recalled: ‘[they]could have told me a little softer that I have a deadly disease’ ID16. Patients reported about experiences where their views were not valued and considered necessary and that healthcare professionals knew their situation better than themselves. One patient told that ‘indifferent encounter-you have to figure things out yourself’ ID9. The staff did not always listen to the patients’ opinions and arguments as on patient expressed: ‘[they] should listen to the patient’s opinion and not immediately knock out’ ID1. The patients’ feelings and experiences of pain, among other things, were asked about, but their answers were not given enough value or taken fully into consideration. Patients felt that they had to wait too long for help, and this was a sign of indifference: ‘indifferent - wait, wait, wait, wait, no matter how painful’ ID9. Another patient expressed the same thing ‘wait - no matter how much your head hurts … while waiting for food you could get medicine, it would be a nicer feeling to eat’ ID1. An extreme example of a patient taking hold of their care expressed their despair by threatening to jump off the balcony if she must wait too long for help. The patient told ‘I said, that I don’t want any 6 hour program, I need help now or I jump from the balcony, I threatened’ ID15.

The loss of a patient´s everyday habits and the repeated need to ask permission for one’s affairs led to *someone taking away one´s self-determination*. According to the patients, small tasks or pleasures that require the permission of others caused feelings of humiliation or helplessness. The patient expressed ‘there’s a strange thing here I’ve never encountered in my life that here we define smoking, for example, and they lock the doors at half past seven’. ID19. Some patients had experiences of power imbalances in the hospital setting when staff can choose the right time and course of action for patients. The patient expressed the matter in different ways such as ‘at 8 a.m. you get coffee, not a moment before’; ‘the need for power - women pretending like it’s the army’; ‘I know myself how much I have to eat due to diabetes, and then the staff determines that no more sandwiches … It is right to be taken away, which I have used the 6 years ...mocking to patronize’; ‘I have taken the right to participate in the decision about my treatment … otherwise it would not have come and will not come’ ID1. Another patient told that ‘the nurse gets to choose when the time is right for them; the patient doesn’t’ D17, which is considered an indirect signal to some patients of their diminished human value. Additionally, the restriction of self-determination was accompanied by an inability to reach loved ones whenever a patient felt to do. One patient asked the interviewer to call his daughter because he could not otherwise contact them. He told that ‘… if you would kindly call my daughter to come here; however, the staff does not call’ ID3.

#### Inadequate Encounters

When speaking of inadequate encounters, it was often a question of what the patient experiences as lacking. Inadequate encounters consisted of two categories: a *lack of presence and lack of support.*

According to the patients, a *lack of presence* was usually a matter of actual time and the quality of the time. Patients often interpreted this as a lack of interest in them. Patients described a physician´s brief conversations about their situation and the nurses’ rapid performance on nursing interventions as ‘a doctor only gives 10–15 minutes of their time, or someone only shows up to ‘change the diapers’’ ID20. The staff did not stop for the patient and be present or ask how they feel. Patients were asked if there was pain, but not how they felt. One patient told that ‘I have been alone, no one has had time to listen to me’ ID1. They hoped the nurses would take the time to talk, not just ask practical questions. One patient said she hoped nurses would ask how they were instead of controlling pain levels or whether they were eating, what she expressed in this way, ‘I wish the nurse would visit here more often; food is brought in and taken away, without asking for anything, never asking how I feel… I would like the nurses to be with me more often’ ID4.

*The lack of support* included lack of informational and emotional support. The lack of informational support was also an urgent topic for patients experiencing negative encounters. Some patients felt ignored concerning information about their disease, treatment and its goal, as one patient described it, ‘I just want to know that plan … I want a plan for what’s worth doing to me’ ID17. Another told that ‘I would like more information about the care plan… I don’t know what is planned’ ID9. A third patient described her situation as the ‘treatment has not been reported. And that’s what I’m wondering, I haven’t even seen a doctor’, and ‘I need more information to tell if anything is being done or not … uncertainty about it’ ID6. A fourth patient said that “these treatments have not been justified for me” ID1. Sometimes the patient felt the lack of information was because even the physician did not know, as one patient described, ‘the doctor themself probably doesn’t even know; they always say they need to make a phone call somewhere’ ID7. The information was not always being presented in a way best suited for them as one patient described ‘I got information sure but only as much as I could understand’ ID12. Another patient described her situation as ‘treatment has not been reported. And that’s what I’m wondering, I haven’t even seen a doctor’, and ‘I need more information to tell if anything is being done or not … uncertainty about it’ ID6. A third patient told that ‘these treatments have not been justified for me’ ID1 and a fourth recanted, ‘I would like more information about the care plan… I don’t know what is planned’ ID9. Sometimes the patient felt the lack of information was because even the physician did not know, as one patient described, ‘the doctor themself probably doesn’t even know; they always say they need to make a phone call somewhere’ ID7. Lack of information also meant that the patient must be active themselves. Patients reported that they had received written information about their treatment, but they had no possibility to discuss it with the staff. One patient said that her tiredness had prevented them from reading the flyers. ‘I have received flyers from all kinds of treatments … but I have not received any information about my own treatment… it would be nice to discuss that’ ID4. They were feeling a loss of control due to a lack of information, and thisled to a distrust of the hospital for some patients.

Some patients felt a lack of emotional support. They felt that the unidentified or unmet needs and support meant that their needs were a problem for healthcare professionals as one patient described ‘healthcare professionals have not supported’ ID4. Another said that ‘I haven’t received support from the health care professionals … they don’t have time, they don’t have enough time’ ID16. One patient stated that he had supported himself when, despite the need, he had not received support from the staff as he described: ‘I have not received the support of the nursing staff; I’ve got myself’ ID1. Another patient said he did not get another appointment with the doctor because his illness was incurable. He was under the impression that the end of care also meant the end of any support from their doctor. He said that ‘the doctor didn’t give me any more time, it says you don’t need it, you have an incurable disease, it gets worse’ ID4. A third patient described that ‘the nurses haven’t talked to me … uurnover is high’ ID5.

## Discussion

According to the findings, the patients receiving palliative care experienced both meaningful and disrespectful encounters in specialist palliative care inpatient units with the healthcare professionals. The patients’ experiences affected their well-being and thus are relevant to the enhancement of their care in the final stage of life. The palliative care patients in this study hoped that healthcare professionals would be present, giving time for the patient through social interaction, and not just limiting the encounter to physical care. Previous studies related to good encounters show similar findings (Kisvetrová et al., 2017; Söderman et al., 2020). In addition, the feeling of being listened to and heard was crucial for patients (Nygårdh et al., 2012).

In this study, an authentic encounter was a type of meaningful encounter. The palliative care patients expressed the importance of being seen as human instead of a patient with generic qualities. Similar findings have come up in previous studies (Akin Korhan et al., 2018; Holopainen et al., 2019; Timmermann et al., 2017). Recognition of humanness, of the self (Söderman et al., 2020), of the patient’s individuality (Nygren Zotterman et al., 2015; 2016) requires holistic care (Akin Korhan et al., 2018; Kmetec et al., 2020). In a palliative care environment and culture, where many practices are technical and medical, the interpersonal interactions were familiar, fundamental and universal human experiences. Appreciating and valuing the patient’s individuality was a meaningful practice, allowing the possibility to self-determine the distance between the healthcare professional and the patient in care situations. Conversations on the future were also hoped for as the patients wished that their future and emotions related to the oncoming death could be discussed. Despite their incurable illness, the patient experienced that they still had a future that should be recognised also by health care professionals. Previous studies have shown that patients want the opportunity to plan and arrange their affairs (Hemberg & Bergdahl, 2020; Houska & Loučka, 2019). Patients in this study also valued conversations concerning the future. Therefore, healthcare professionals should encourage the patient to openly reflect and discuss the future(Houska & Loučka, 2019; Söderman et al., 2020; Timmermann et al., 2017). Meaningfulness in encounters is possible if the healthcare professionals have enough sensitivity to understand and sense the situation from the patient perspective (Wälivaara et al., 2013). Patients’ needs for dignity, individuality and privacy should be identified (Karbasi et al., 2018) and the distinctive nature of the everyday situations recognised (Haavisto et al., 2020).

The interviewees experienced supportive encounters by having information needs identified and by being able to ask questions. However, the patients did not want to know too much. They trusted the physician to know the level of information to be provided. Patients in a previous study also mentioned conversations related to disease and treatment (Chua et al., 2020). Further, it has been found that patients are often expected to get a broad picture of what to expect, not detailed information (Chua et al., 2020; Hemberg & Bergdahl, 2020). However, this study found that there were gaps in the process of informing patients. Some patients felt that they were completely ignored when it came to inform them about their treatment. Patients also emphasised advocacy (i.e. the experience of having your care providers on your side). The significance of healthcare professionals, mainly the nurses acting as an advocate to the patient, has been perceived to be significant (Karbasi et al., 2018).

According to previous studies, uncaring encounters can cause negative emotions (Hemberg & Bergdahl, 2020; Holopainen et al., 2019), leaving patients feeling that they have received deficient and unempathetic care (Cheruiyot & Brysiewicz, 2019). Patients in this study experienced disrespectful encounters with others in the form of indifferent and inadequate encounters. These are accounts of encounters where the interaction did not involve an equal and interpersonal connection (Wälivaara et al., 2013). Patients felt that their views and opinions were not appreciated, and they were ignored, which can lead to the experience that patients are excluded from their own care and life (Jørgensen et al., 2019). However, several studies have shown that participation in their own care is important to patients (Houska & Loučka, 2019; Nygren Zotterman et al., 2015). Another issue that the patients described in this study as indifferent are the feelings of someone taking away their self-determination. Emotions of exclusion can lead to the feeling that the patients’ fundamental needs are not being met (Holopainen et al., 2019; Nygårdh et al., 2012).

The dying patients in this study hoped that healthcare staff would be more present, that they would have more time for the patient and that they would also discuss, not just perform. Nygård and colleagues stated that the feeling of being listened to and heard is crucial for patients (Nygårdh et al., 2012). However, one of the dying patients in this study expressed the importance of self-determined alone time, which has not been shown in previous studies.

Some of the interviewed dying patients also experienced an absence of support and of physical contact. According to Nygren Zotterman and colleagues, being treated with carelessness, distance and nonchalance by the healthcare staff can make patients feel meaningless and insignificant (Nygren Zotterman et al., 2016).

### Study Limitations

There are some limitations related to this study. First, it should be noted that not all eligible patients who showed interest in describing their experiences were reached. Patients who were confused, anxious or in poor physical condition were assessed as unsuitable for the study. Second, the number of interviewees consisted of 20 patients, which is a suitable amount for the content analysis method and the saturation of the data was attained through reoccurrence of the themes. The high number was evaluated as justified since some interviews were brief due to the interviewees’ poor health. No transcripts were returned to the interviewees for corrections or the possibility of giving feedback about the results because weakening condition of the patients. Third, the interviews were conducted by two researchers, which might influence the consistency of the data collection. However, the predefined themes were followed during the interviews to maintain the consistency of the interviews. Fourth, data analysis was conducted by two authors, and the findings were discussed as well as reviewed together with the research group to support trustworthiness and confirmability. Finally, consolidated criteria for reporting qualitative research (COREQ) supported study reporting (Tong et al., 2007).

## Conclusions

The study describes how palliative care cancer patients vocalise their needs to be treated with dignity through a sense of control over one’s care, self-determination and equality. In palliative care, encounters should not only be seen as situations related to discussion and information sharing but as a holistic consideration of patients and a demonstration of humanity. Caring for a palliative care patient requires care beyond tending to a patient’s physical needs. The social encounters between a healthcare professional and a patient can significantly affect a patient’s experiences of palliative care. Most patients long to be seen as human beings with value and individualised lived experiences. Several patients experience the encounters with their healthcare professionals as a direct reflection of their human worth. Healthcare professionals should recognise the need for genuine encounters with palliative care patients.

Furthermore, patients should be encountered holistically and as equal human beings without highlighting their roles as patients. The healthcare professionals and the organisations should also acknowledge the importance of time and effort spent for encounters and discussions with the patients instead of concentrating resources mainly on physical care. For future research, a corresponding study from the perspectives of both healthcare staff and the family members would also be of value in examining encounters.
